# Personal

**Published:** 1891-09

**Authors:** 


### Personal.

Dr. George H. Rohé, of Baltimore, has resigned as Health Officer of that city to accept the Superintendency of a Hospital for the Insane near Baltimore.

Dr. L. Duncan Bulkley, of New York, has been awarded the Alvarenga Prize of the College of Physicians of Philadelphia for 1891, for an essay on Syphilis Insontium.

Prof. John A. Miller, Ph. D., of Buffalo, was elected a member of the Executive Committee of the American Microscopical Society, at its recent meeting held in Washington, D. C.

Dr. F. B. Darling, formerly of Westfield, N. Y., has located at 273 Franklin street, Buffalo, for the practice of his profession. His office hours are: 8 to 10 a. m., 2 to 5, and 7 to 9 p. m.

Dr. Russell Bellamy, of Wilmington, N. C., won the "Appleton Prize," in Asheville, last month, he having stood the best examination out of eighty applicants for license before the State Examining Board.

H. M. Whelpley, Ph. G., M. D., F. R. M. S., has been elected Professor of Physiology and Histology, and Director of the Histological Laboratory of the Missouri Medical College, St. Louis, as well as Secretary of the faculty.

Dr. E. E. Montgomery, of Philadelphia, spent the month of August on the Pacific coast, where he attended a number of medical society meetings, thus combining professional work and recreation in a most agreeable manner. He was accompanied by Mrs. Montgomery.

Dr. John H. Rauch, of Chicago, has tendered his resignation as Secretary of the Illinois State Board of Health. The medical profession of the United States owes Dr. Rauch an everlasting debt of gratitude for his services in the interest of higher medical education. He has waged an unrelenting warfare against all forms of ignorance and irregularity in medicine for many years, and his retirement from his labors is to be regretted. But it must be confessed that he has well earned his much-needed rest, and we wish him long years of enjoyment, with his blushing honors so thick upon him.

---

A Good Selection.—Gen. James A. Beaver, late Governor of Pennsylvania, a scholar, a lawyer, a soldier, and a man of fine attainments, has recently been selected as President of the Board of Trustees of the Philadelphia Dental College in place of the late Governor Pollock. General Beaver, who has always taken so much interest in the scientific, educational and charitable institutions of this State, will make an able head to this worthy school. The college at this time is one of the best equipped, and has one of the most distinguished lists of teachers in the world. Large numbers of students from every portion of the civilized world annually seek the halls of this college.—*Medical Bulletin.*

